# Retraction: Application of cytology and molecular biology in diagnosing premalignant or malignant oral lesions

**DOI:** 10.1186/1476-4598-11-57

**Published:** 2012-08-20

**Authors:** Ravi Mehrotra, Anurag Gupta, Mamta Singh, Rahela Ibrahim

**Affiliations:** 1 Mahamaya Government Allopathic Medical College, Ambedkarnagar, Uttar Pradesh, India; 2 S.N. Medical College, Agra, India; 3 Moti Lal Nehru Medical College Allahabad, Ambedkarnagar, Uttar Pradesh, India

## Retraction

The authors would like to retract the article "Application of cytology and molecular biology in diagnosing premalignant or malignant oral lesions" [1]. After publication of the article it came to light that large portions of the article are duplicated from a previously published article [2]. The authors apologize to the readers, reviewers, and Editors of *Molecular Cancer* for the inconvenience caused.
